# Purple Phototrophic Bacterium Enhances Stevioside Yield by *Stevia rebaudiana* Bertoni via Foliar Spray and Rhizosphere Irrigation

**DOI:** 10.1371/journal.pone.0067644

**Published:** 2013-06-25

**Authors:** Jing Wu, Yiming Wang, Xiangui Lin

**Affiliations:** 1 State Key Laboratory of Soil and Sustainable Agriculture, Institute of Soil Science, Chinese Academy of Sciences, Nanjing, People’s Republic of China; 2 Joint Open Laboratory of Soil and the Environment, Hong Kong Baptist University & Institute of Soil Science, Chinese Academy of Sciences, Nanjing, People’s Republic of China; 3 University of Chinese Academy of Sciences, Beijing, People’s Republic of China; BASF Cropdesign, Belgium

## Abstract

This study was conducted to compare the effects of foliar spray and rhizosphere irrigation with purple phototrophic bacteria (PPB) on growth and stevioside (ST) yield of *Stevia. rebaudiana*. The *S. rebaudiana* plants were treated by foliar spray, rhizosphere irrigation, and spray plus irrigation with PPB for 10 days, respectively. All treatments enhanced growth of *S. rebaudiana*, and the foliar method was more efficient than irrigation. Spraying combined with irrigation increased the ST yield plant ^-1^ by 69.2% as compared to the control. The soil dehydrogenase activity, *S. rebaudiana* shoot biomass, chlorophyll content in new leaves, and soluble sugar in old leaves were affected significantly by S+I treatment, too. The PPB probably works in the rhizosphere by activating the metabolic activity of soil bacteria, and on leaves by excreting phytohormones or enhancing the activity of phyllosphere microorganisms.

## Introduction

*Stevia rebaudiana* Bertoni is a herb native to certain regions of South America. It produces several valuable and sweet steviol glycosides in its leaves [1]. The dried leaves have been used as natural sweeteners for centuries in some countries. Regulatory authorities worldwide have permitted the use of the steviol glycosides extracted from *S. rebaudiana* leaves as a dietary supplement. Stevioside (ST), one of the steviol glycosides, is 300 times sweeter than cane sugar and non-calorific, and has specific immunomodulatory activities [2], [3], leading to its use as a sweetener by diabetic and phenylketonuria patients and obese persons. Therefore, *S. rebaudiana* is widely planted because of the great demand for ST to be used in products targeted at those specific consumers. A number of research projects have tried to improve the *S. rebaudianas* growth and ST yield using component fertilizers or plant growth regulators [4]–[6]. It has also been reported that increasing chlorophyll content could directly affect steviol glycosides yield [7], and there is a significant positive correlation between total sugar content and ST content [8]. Contents of chlorophyll and total sugar are correlated to photosynthesis. Thus, enhancing photosynthesis may be a way to help increase ST production.

Microorganisms also affect the growth of *S. rebaudiana* [9], [10]. Maintenance of bacteria–plant interactions has been suggested as a effective strategy to improve plant productivity. For example, purple phototrophic bacteria (PPB) interact with and benefit plants, and have been applied to crops such as rice, tomatoes, and mushrooms for many years [11]–[13]. However, the effects and processes of interaction between plants and PPB are still under investigation. Some studies reported that PPB contain carotenoid pigments and biological cofactors, and release secondary metabolites, such as phytohormones, to promote plant growth [14], [15]. Others suggested that PPB facilitate the turnover and transformation of organic matter in soil through enhancing the metabolic activity of soil microorganisms [16], [17]. As soil dehydrogenase plays an essential role in the initial stages of the oxidation of soil organic matter by transferring hydrogen and electrons from substrates to acceptors, it has been widely used to measure total metabolic activity of soil microorganisms [18]. For example, optimizing and balancing applications of fertilizers or bio-fertilizers leads to a significant increase in dehydrogenase activity [19].

However, the soil environment lacks light, and thus differs from the laboratory culture conditions of PPB. Light has been shown to be important to the growth and metabolism of PPB, and physiological processes may be inhibited if there is no light [20]. Foliar application of microorganisms avoids many of the biotic and abiotic factors and constraints of the soil environment [21]. Many studies have indicated that foliar application of bacteria suspensions can promote growth of plants [22]. When leaves are sprayed with PPB, the bacteria survive with light which is different from with dark in soil. Nevertheless, whether foliar application of PPB can result in plant-growth-promoting effects has been poorly studied [23].

To date, there have been no investigations into the effects of PPB by irrigation or by spraying on growth and ST yield of *S. rebaudiana*. The objective of the present study is to compare the effects of foliar spray and/or rhizosphere irrigation of PPB on *S. rebaudiana* and to identify the key factors that influence ST yield.

## Materials and Methods

### Preparation of Bacterial Suspensions

The test PPB strain *Rhodopseudomonas* sp. (ISP-1) was isolated from Xuanwu Lake, Nan Jing, Jiangsu Province, China. It was grown in a waste water medium: 250 mL tofu processing wastewater plus 750 mL water per 1 L medium. The pH of the medium was adjusted to 7.0 before sterilization. The bacterial culture was incubated at 28±4°C under a 60 W incandescent lamp at a distance of 25 cm for 7 days. PPB cells were extracted by centrifugation and washed twice by resuspension in a 0.9 g L^−1^ NaCl solution followed by centrifugation. Then, the cells were resuspended and diluted with sterile water to optical density 0.8 at 600 nm, which corresponded to a bacterial density of 3×10^10^ active PPB cells mL^−1^ by the plate count method. This suspension was designated suspension 1. The suspension 2, 3, and 4 were 10%, 20%, and 50% dilutions of suspension 1. These suspensions were prepared to treat *S. rebaudiana*.

### Soil and *S. rebaudiana* Preparation

This study was a greenhouse experiment. The soil samples were supplied kindly by Zhucheng Haotian Pharm Co. Ltd. from land used to grow *S. rebaudiana* in Zhucheng, Shandong Province, China (36°4′20′′N; 119°34′53′′E). There were no endangered or protected species in the location where the soil was collected. No specific permits were required for the study. The collected soil had a pH of 7.4 (soil: water ratio of 1∶2.5), contained 9.5 g kg^−1^ of organic C, and 10.32, 36.08, and 205.60 mg kg^−1^ of available N, P, and K, respectively. The soil was air dried and then passed through a 2-mm sieve to remove root debris and large stones.

A high-stevioside-yielding variety of *S. rebaudiana* was used in this experiment. It has already been screened and put into mass production by Zhucheng Haotian Pharm Co. Ltd. Thirty shoot tips with three leaves were collected as explants from the chosen *S. rebaudiana*. The explants were rooting in sterile sand for 15 days and then transplanted into pots, with one plant per pot. After 75 days’ growth in pots, 12 plants with similar growth status were selected and divided into four treatment groups with three replicates in a completely randomized design.

### Pot Experiment and Plant Harvest

The experiment was carried out in the greenhouse of the Institute of Soil Science, Chinese Academy of Science, Nanjing, China. There were four treatments: 1) foliar spray (S): *S. rebaudiana* leaves were sprayed with 50 mL PPB suspension 3 once a day for eight days, and irrigated once with 100 mL of sterile water; 2) rhizosphere irrigation (I): the soil in the pot was irrigated with 100 mL suspension 1 once, and the leaves sprayed with 50 mL sterile water once a day for eight days; 3) spray+irrigation (S+I): the leaves were sprayed with 50 mL suspension 2 eight times and the soil was irrigated with 100 mL of suspension 4; and 4) the control (CK): plants were sprayed and irrigated with sterile water.

The *S. rebaudiana* plants were harvested after being treated for 10 days. All plant shoots were harvested. Two fully expanded mature leaves from the four most basal nodes (old leaves) and two developing leaves from the four uppermost apical nodes (new leaves) were collected, washed, and weighed before assessing the chlorophyll and total soluble sugar content. Shoots were weighed after oven drying at 45°C for 96 h, and seperated leaves from stems. The *S. rebaudiana* leaves were ground to powder to determine ST concentration. Fresh soil samples were collected from pots to analyze dehydrogenase activity.

### Analyses of Chlorophyll, Soluble Sugar, and Stevioside

Chlorophyll extraction was performed by homogenization of 0.5 g fresh leaf material with 50 ml 80% acetone, and the contents of chlorophyll a and b were calculated according to the method of Arnon [24]. The soluble sugar was measured by homogenization of 0.2 g fresh leaves in 10 mL of deionized water by heating at 100°C for 30 min. The extraction was repeated three times. Supernatants were filtered, collected, and made up to a total volume of 50 mL. Contents of soluble sugar were determined according to the method described by Ding et al. [25]. ST in the dry leaf material was measured by the high performance liquid chromatography (HPLC) method. To extract the ST, 1 g of ground leaf material was heated in 25 ml deionized water at 100°C for 1 hour; the extraction was repeated three times. Supernatants were filtered, collected, and made up to a total volume of 100 ml. The ST content was determined by the Prominence UFLC C-18 column (250 mm × 46 mm, 5 µm, Shimadzu, Japan), in the ascending mode in a methanol–water (68∶32) solvent system. The eluent flow rate velocity was 0.9 mL min^−1^. The wavelength of UV detector was 203 nm [26]. The standard reference material for ST was purchased from the National Institute for Food and Drug Control (HPLC≥99%).

### Analysis of Soil Dehydrogenase Activity

Fresh soil was homogenized by passing it through a 2-mm sieve, and then dehydrogenase activity was determined by the reduction of triphenyltetrazolium chloride (TTC) to triphenylformazan (TPF) as described by Serra-Wittling *et al*. [27]. This result was expressed on an oven-dried soil weight basis (105°C, 24 h).

### Statistical Analysis

All results were calculated by SPSS version 13.0 for Windows. Significant differences of means for all treatments were calculated using Duncan’s multiple range tests (*P*<0.05). Redundancy analysis (RDA) was calculated by Canoco version 4.5 to elucidate the relationships between *S. rebaudiana* parameters, soil dehydrogenase activity, and bacterial treatments.

## Results

### Soil Dehydrogenase Activity, Chlorophyll a/b Content and Soluble Sugar in *S. rebaudiana* Leaves

The soil dehydrogenase activity was increased by either foliar spray (S) or rhizosphere irrigation (I) as compared to the control, and significantly elevated (*P*<0.05) by the combined treatment of spray and irrigation (S+I) (Table 1). The treatment S significantly increased (*P*<0.05) chlorophyll a and b content in old leaves, and increased chlorophyll a, b and soluble sugar content in old leaves, but had no effect on soluble sugar content in new leaves and chlorophyll b content in new leaves. The treatment I also increased chlorophyll a and b content in old leaves, and incresed soluble sugar content in new leaves, but had no effects on soluble sugar content in old leaves and chlorophyll a and b content in new leaves. The combined treatment S+I significantly increased (*P*<0.05) chlorophyll a and b content in old leaves and chlorophyll a content in new leaves compared to both CK and I, and significantly increased (*P*<0.05) soluble sugar content in new leaves compared to the CK, but still had no effect on either soluble sugar content in old leaves or chlorophyll b content in new leaves.

**Table 1:** Soil dehydrogenase activity, leaf chlorophyll a, chlorophyll b, and soluble sugar contents.

Treatments	Soil (mg TPF kg^−1^ dw d^−1^)	Old leaf (mg g^−1^FW)			New leaf (mg g^−1^FW)		
	Dehydrogenase activity	Chlorophyll a	Chlorophyll b	Soluble sugar	Chlorophyll a	Chlorophyll b	Soluble sugar
CK	13.10±2.74b	1.48±0.26c	0.86±0.10c	22.97±1.60b	1.11±0.078b	0.64±0.049a	31.98±3.43a
S	17.41±1.81ab	2.22±0.22ab	1.26±0.081ab	40.69±12.37ab	1.4±0.26ab	0.81±0.16a	30.68±9.78a
I	18.41±3.74ab	1.92±0.45bc	1.11±0.27bc	36.19±11.48ab	1.19±0.18b	0.70±0.10a	33.52±9.08a
S+I	20.81±2.86a	2.82±0.44a	1.56±0.24a	49.59±9.52a	1.46±0.14a	0.82±0.075a	31.57±6.77a

S: spray PPB; I: irrigation PPB; S+I: spray+irrigation PPB; CK: control. standard deviation of the mean (n = 3) is shown, different letters demonstrate a significant difference at *P*<0.05.

### *S. rebaudiana* Shoot Biomass, Stevioside Content in Leaf, and Stevioside Yield

**Figure 1:**
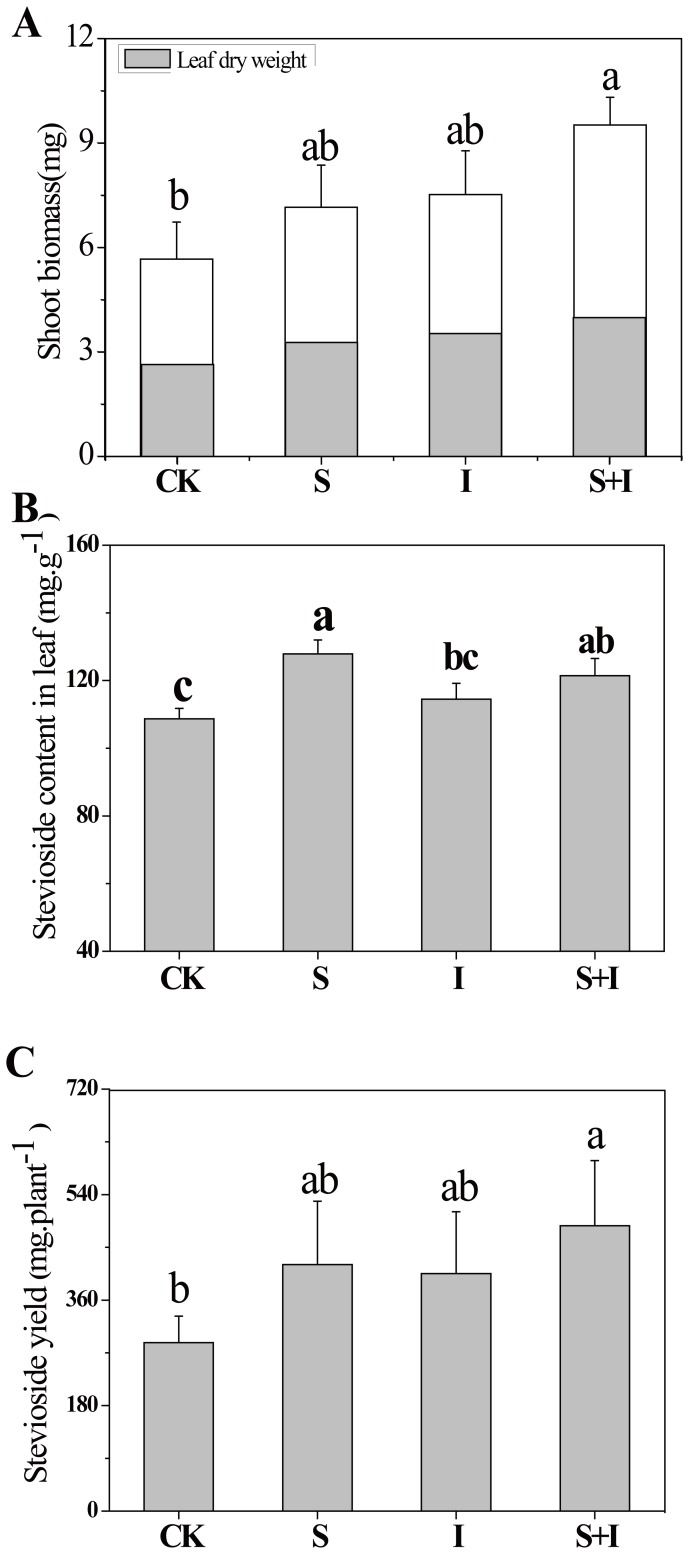
Plant biomass (a), stevioside content (b), and stevioside yield (c) of *S. rebaudiana* under different PPB treatments. S: spray PPB; I: irrigation PPB; S+I: spray+irrigation PPB; CK: control. Bars indicate the standard deviation of the mean (n = 3), different letters demonstrate a significant difference at *P*<0.05.

The effect of PPB on shoot biomass, ST content both on per gram dry leaf weight as well as per plant dry leaf weight of *S. rebaudiana* was observed. Compared to CK, both treatments S and I elevated *S. rebaudiana* shoot biomass (Figure 1A) and the total ST yields per plant (Figure 1C). The treatment S significantly increased (*P*<0.05) ST content in *S. rebaudiana leaves* (Figure 1B), but treatment I had no similar effect. Compared to CK, the combined treatment (S+I) hugely elevated (*P*<0.05) *S. rebaudiana* shoot biomass and the total ST yield per plant, but the leaf ST content was not increased significantly.

### Redundancy Analysis of *S. rebaudiana* Parameters, Soil Dehydrogenase Activity, and PPB Treatments

**Figure 2:**
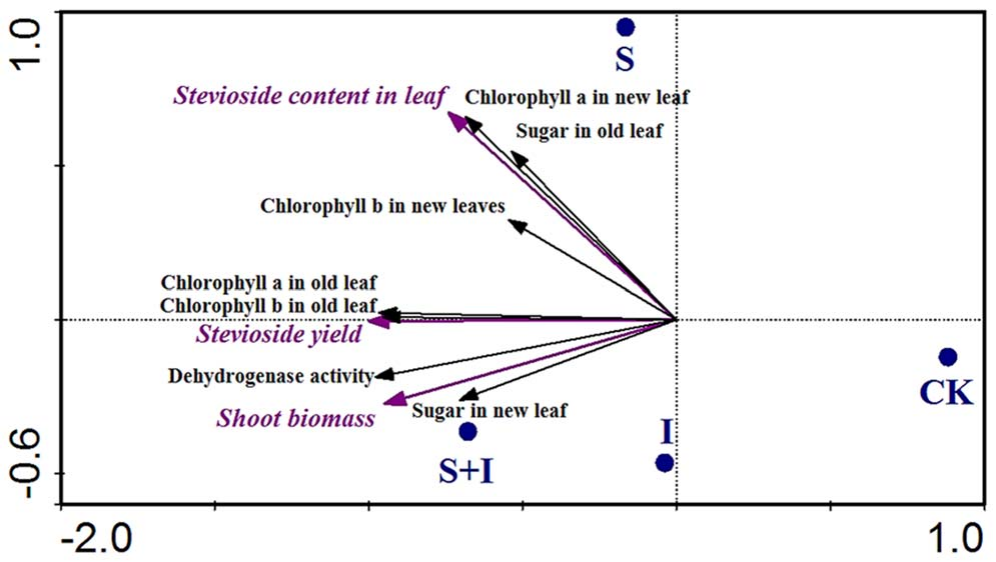
RDA of *S. rebaudiana* parameters and soil dehydrogenase activity under different PPB treatments. S: spray PPB; I: irrigation PPB; S+I: spray+irrigation PPB; CK: control.

In the RDA plot, projecting an object at a right angle on a response variable approximates the value of the object along that variable, while the angles between response variables themselves reflect their correlations. The RDA result of *S. rebaudiana* was shown in Figure 2. Compared with CK and I, S had the greatest effect on ST content in leaves, which correlated to chlorophyll a content in new leaves and sugar in old leaves (r = 0.750, *P*<0.01; r = 0.579, *P*<0.05). The combined treatment (S+I) had the greatest effects on shoot *S. rebaudiana* biomass, which significantly correlated to sugar in new leaves and soil dehydrogenase activity (r = 0.687, and 0.651, *P*<0.05). In addition, the ST yield per plant was also greatly enhanced by the combined treatment (S+I) and related closely to content of chlorophyll a/b in old leaves (r = 0.708, *P*<0.01 and r = 0.651, *P*<0.05).

## Discussion

The soil dehydrogenase activity is considered to be an indicator of soil microbial activity [28]. Greater metabolic activity of soil bacteria makes more nutrients available to plants. In this study, the irrigation of rhizosphere with PPB (I) apparently promoted soil dehydrogenase activity, increased chlorophyll a and b contents in old leaves, and then slightly enhanced shoot biomass of *S. rebaudiana*. Thus, soil dehydrogenase activity correlated markedly to shoot biomass (Figure 2). The roles of soil application of PPB in the improvement of plant shoot biomass have been previously demonstrated [9], [29]. The observations in the present study are consistent with reports that the shoot and root biomass of tomato increased after irrigation with a suspension of *Rhodopseudomonas* sp. [12].

The role of foliar spray of plant-growth-promoting bacteria in improving growth of mulberry, apricot, and wheat has been reported by Sudhakar et al., Estiken et al., and Ebrahim et al. respectively [30]–[32]. In this study, chlorophyll a and soluble sugar contents of new leaves were increased with the spray treatment (S). It is noteworthy that the relationships among chlorophyll, sugar, and ST were positive. The foliar spray had a similar effect to that shown in the study of the effect of *Rhodopseudomonas* sp. suspension on mushroom cultures: spraying the bacterial suspension on casing soil and between the flushes could significantly increase the mushroom yield [13]. Yin et al. also found that the fresh weight of sweet cherry was increased by foliar spraying with PPB suspension [23]. Contents of chlorophyll and soluble sugar were correlated with the intensity of photosynthesis. It was suggested that spraying with PPB might activate *S. rebaudiana* photosynthesis, which in turn could stimulate plant growth and increase ST concentration according to Jain et al. and Metiver and Viana [7], [8]. A number of studies reported that purple non-sulfur bacteria could accumulate and excrete large amounts of 5-amino levulinic acid extracellularly, thus leading to a rise in chlorophyll content and photosynthesis by plants following foliar application [33], [34]. In addition, it has been reported recently that the phyllosphere contains a phylogenetically diverse assemblage of phototrophic species, including anoxygenic phototrophic bacteria, and these species were capable of harvesting light [35]. Providing additional PPB on leaves might increase the populations of the phyllosphere microorganisms or activate them. Thus, the ratio of chlorophyll a to b of leaves was increased by foliar spraying with PPB suspension; this is a signal of the leaves’ capacity to capture solar energy and a method to boost the photosynthesis of stevia. In theory, foliar application of bacterial spray may indeed be an environmentally friendly fertilization method and involve reduced competition from other microorganisms and environmental factors compared to rhizosphere irrigation.

Moreover, the effect of combination (S+I) treatment was the most obvious because it induced the greatest increase in the *S. rebaudiana* shoot biomass (68.3%). Harada et al. found that the yield of rice can increase by 29% if suitable phototrophic purple non-sulfur bacteria are inoculated into the paddy soil [11]. Meanwhile, PPB application can also improve productivity in terms of production and biochemical constituents. In this study, the S+I treatment enhanced ST yield by 69.2%. This is the first study to show an increase in ST yield by the PPB treatment. A similar increase in ST yield was found in plants inoculated with *Serratia marcescens* 10238 and *Burkholderia gladioli* 10216 [10]. Although foliar sprays could be used as supplements for soil application, they cannot be a substitute for soil fertilization in all cases due to the existing lack of knowledge on the penetration of the leaf-applied solutions. The combination of foliar spray and rhizosphere irrigation of PPB could result in remarkable effects on both *S. rebaudiana* growth and ST yield. A field trial is still needed for better understanding of how this method could be used in practice and the mechanisms involved.

### Conclusions

All PPB treatments enhanced growth of *S. rebaudiana*, and foliar spray was more effective than rhizosphere irrigation. Spray combined with irrigation greatly increased the yield of stevioside (69.2%) compared with the control. Thus, it is a practical and effective way to improve ST yield. Soil dehydrogenase activity, shoot biomass, chlorophyll content in new leaves, and soluble sugar in old leaves were affected significantly by the combined method. In soil, PPB probably activated the metabolism of soil bacteria, and thus, plant growth was promoted by this activity in the rhizosphere; on leaves PPB probably excreted phytohormones or enhanced activity of phyllosphere microorganisms, and thus the plant increased photosynthetic activity and produced more stevioside.

## References

[1] Salerno G, Curatti L (2003) Origin of sucrose metabolism in higher plants: when, how and why? Trends Plant Sci 8: 63–69. 12597872 10.1016/S1360-1385(02)00029-8

[2] Chatsudthipong V, Muanprasat C (2008) Stevioside and related compounds: Therapeutic benefits beyond sweetness. Pharmacol Ther 121: 41–54. 19000919 10.1016/j.pharmthera.2008.09.007

[3] Boonkaewwan C, Ao M, Toskulkao C, Rao MC (2008) Specific immunomodulatory and secretory activities of stevioside and steviol in intestinal cells. J Agric Food Chem 56: 3777–3784. 18433103 10.1021/jf072681o

[4] Das K, Dang R, Shivananda TN (2006) Effect of N, P and K fertilizers on their availability in soil in relation to the Stevia plant (*Stevia rebaudiana* Bert.) Arch Agr Soil Sci. 52: 679–6850.

[5] Ma L, Shi Y (2011) Effects of potassium fertilizer on physiological and biochemical index of *Stevia rebaudiana* Bertoni. Energy Procedia 5: 581–586.

[6] Ren GX, Liu XY, Shi Y (2011) Effects of plant growth regulator S-Y on diurnal changes in photosynthetic parameters and yield of *Stevia Rebaudina* Bertoni. Energy Procedia 5: 429–434.

[7] Jain P, Sumita K, Kothari SL (2009) Improved micropropagation protocol and enhancement in biomass and chlorophyll content in *Stevia rebaudiana* (Bert.) Bertoni by using high copper level in the culture. Sci Hort 119: 315–319.

[8] Metivier J, Viana AM (1979) The effect of long and short day length upon the growth of whole plants and the level of soluble protiens, sugars and stevioside in leaves of *Stevia rebaudiana* . J Exp Bot 30: 1211–1222.

[9] Das K, Dang R, Shivananda TN, Sekeroglu N (2007) Influence of bio-fertilizers on the biomass yield and nutrient content in Stevia (*Stevia rebaudiana* Bert.) grown in Indian subtropics. J Med Plants Res 1: 5–8.

[10] Mamta RP, Pathaniad V, Gulatic A (2010) Stimulatory effect of phosphate solubilizing bacteria on plant growth, stevioside and rebaudioside- a contents of *Stevia rebaudiana* Bertoni. Appl Soil Ecol 46: 222–229.

[11] Harada N, Nishiyama M, Otsuka S, Matsumoto S (2005) Effects of inoculation of phototrophic purple bacteria on grain yield of rice and nitrogenase activity of paddy soil in a pot experiment. Soil Sci. Plant Nutr 51: 361–367.

[12] Lee KH, Koh RH, Song HG (2008) Enhancement of growth and yield of tomato by *Rhodopseudomonas sp.* under greenhouse conditions. J Microbiol 46: 641–646. 19107392 10.1007/s12275-008-0159-2

[13] Han JR (1999) The influence of photosynthetic bacteria treatments on the crop yield, dry matter content, and protein content of the mushroom *Agaricus bisporus*. Sci Hort. 82: 171–178.

[14] Pfenning N (1967) Photosynthetic bacteria. Ann Rev Microbiol 21: 285–324. 4860261 10.1146/annurev.mi.21.100167.001441

[15] Rajasekhar N, Sasikala Ch, Ramana ChV (1999) Photoproduction of indole 3-acetic acid by *Rhodobacter sphaeroides* from indole and glycine. Biotechnol Lett 21: 543–545.

[16] Liang CM, Hung CH (2010) Purple nonsulfur bacteria diversity in activated sludge and its potential phosphorus-accumulating ability under different cultivation conditions. Appl Microbiol Biotechnol 86: 709–719. 19943045 10.1007/s00253-009-2348-2

[17] Ludden PW, Roberts GP (2002) Nitrogen fixation by photosynthetic bacteria. Photosynth Res 73: 115–118. 16245111 10.1023/A:1020497619288

[18] Ross DJ (1971) Some factors influencing the estimation of dehydrogenase activities of some soils under pasture. Soil Biol Bioch 3: 97–110.

[19] Chang EH, Chung RS, Tsai YH (2007) Effect of different application rates of organic fertilizer on soil enzyme activity and microbial population. Soil Sci Plant Nutr 53: 132–140.

[20] Sasikala K, Ramana CV, Rao PR (1991) Environmental regulation for optimal biomass yield and photoproduction of hydrogen by *Rhodobacter* sphaeroides O.U.001. Int J Hydrogen Energy16: 597–601.

[21] Javaid A (2010) Beneficial microorganisms for sustainable agriculture. In: Lichtfouse E, editor. Genetic engineering, biofertilisation, soil quality and organic farming. Dordrecht: Springer Publishers. 347–369.

[22] Esitken A, Pirlak L, Turen M (2006) Effects of floral and foliar application of plant growth promoting rhizobacteria (PGPR) on yield, growth and nutrition of sweet cherry. Sci Hort 110: 324–327.

[23] Yin ZP, Shang ZW, Wei C, Ren Jing, Shun X (2012) Foliar sprays of photosynthetic bacteria improve the growth and anti-oxidative capability on Chinese dwarf cherry seedings. J Plant Nutr 35: 840–853.

[24] Arnon DI (1949) Copper enzymes in isolated chloroplasts. Polyphenoloxidase in *Beta vulgaris* . Plant Physiol 24: 1–15. 16654194 10.1104/pp.24.1.1 PMC437905

[25] Ding Y, Luo W, Xu G (2006) Characterisation of magnesium nutrition and interaction of magnesium and potassium in rice. Ann Appl Biol 149: 111–123.

[26] Kedik SA, Fedorov SV, Yanul NA (2003) Chromatographic determination of stevioside in raw plant material. Pharma Chem 37: 529–532.

[27] Serra-Wittling C, Houot S, Barriuso E (1995) Soil enzymatic response to addition of municipal solid-waste compost. Biol FertSoils 20: 226–236.

[28] Tabatabai MA (1994) Soil enzymes. In: Weaver RW, Angle JS, Bottomley PS (eds) Methods of soil analysis. part II. Microbiological and biochemical properties, 5. Soil Science Society of America, Madison, Wis, 775–833.

[29] Shi QL, Yang SP, Ma YZ, Zhang ZM (1995) The effect of nutritional liquid manure of active PSB on grape. J Shanghai Jiaotong University 18: 329–331 (in Chinese with English abstract).

[30] Sudhakar P, Chattopadhyay GN, Gangwar SK, Ghosh JK (2000) Effect of foliar application of *Azotobacter*, *Azospirillum* and *Beijerinckia* on leaf yield and quality of mulberry (*Morus alba*). J Agr Sci 134: 227–234.

[31] Esitken A, Karlidag H, Ercisli S, Sahin F (2002) Effects of foliar application of *Bacillus subtilis* Osu-142 on the yield, growth and control of shot-hole disease (Coryneum blight) of apricot. Gartenbauwissenschaf 67: 139–142.

[32] Ebrahim MKH, Aly MM (2004) Physiological response of wheat to foliar application of zinc and inoculation with some bacterial fertilizers. J Plant Nutr 27: 1859–1874.

[33] Hotta Y, Tanaka T, Takaoka H, Takeuchi Y, Konnai M (1997a) New physiological effects of 5-aminolevulinic acid in plants: The increase of photosynthesis, chlorophyll content, and plant growth. Biosci. Biotech. Biochem 61: 2025–2028. 10.1271/bbb.61.2025 27396878

[34] Sasaki K, Tanaka T (1991) Enhanced production of 5-aminolevulinic acid by repeated addition of levulinic acid and supplement of precursors in photoheterotrophic culture of *Rhodobacter sphaeroides* . J Fermentation Technol 71: 403–406.

[35] Finkel OM, Burch AY, Lindow SE, Post AF, Belkin S (2011) Phyllosphere microbial communities of a salt-excreting desert tree: geographical location determines population structure. Appl Environ Microbiol 77: 7647–7655. 21926212 10.1128/AEM.05565-11 PMC3209174

